# The Complexity and Multiplicity of the Specific cAMP Phosphodiesterase Family: PDE4, Open New Adapted Therapeutic Approaches

**DOI:** 10.3390/ijms231810616

**Published:** 2022-09-13

**Authors:** Claire Lugnier

**Affiliations:** Section de Structures Biologiques, Pharmacologie et Enzymologie, CNRS/Unistra, CRBS, UR 3072, CEDEX, 67084 Strasbourg, France; claire.lugnier@unistra.fr; Tel.: +33-(0)6-8422-2454

**Keywords:** cyclic nucleotide phosphodiesterase (PDE), cAMP, cGMP, selective PDE4 inhibitor (PDE4-I), interactome, inflammation, cancer, COVID-19

## Abstract

Cyclic nucleotides (cAMP, cGMP) play a major role in normal and pathologic signaling. Beyond receptors, cyclic nucleotide phosphodiesterases; (PDEs) rapidly convert the cyclic nucleotide in its respective 5′-nucleotide to control intracellular cAMP and/or cGMP levels to maintain a normal physiological state. However, in many pathologies, dysregulations of various PDEs (PDE1-PDE11) contribute mainly to organs and tissue failures related to uncontrolled phosphorylation cascade. Among these, PDE4 represents the greatest family, since it is constituted by 4 genes with multiple variants differently distributed at tissue, cellular and subcellular levels, allowing different fine-tuned regulations. Since the 1980s, pharmaceutical companies have developed PDE4 inhibitors (PDE4-I) to overcome cardiovascular diseases. Since, they have encountered many undesired problems, (emesis), they focused their research on other PDEs. Today, increases in the knowledge of complex PDE4 regulations in various tissues and pathologies, and the evolution in drug design, resulted in a renewal of PDE4-I development. The present review describes the recent PDE4-I development targeting cardiovascular diseases, obesity, diabetes, ulcerative colitis, and Crohn’s disease, malignancies, fatty liver disease, osteoporosis, depression, as well as COVID-19. Today, the direct therapeutic approach of PDE4 is extended by developing allosteric inhibitors and protein/protein interactions allowing to act on the PDE interactome.

## 1. Introduction

**Figure 1:**
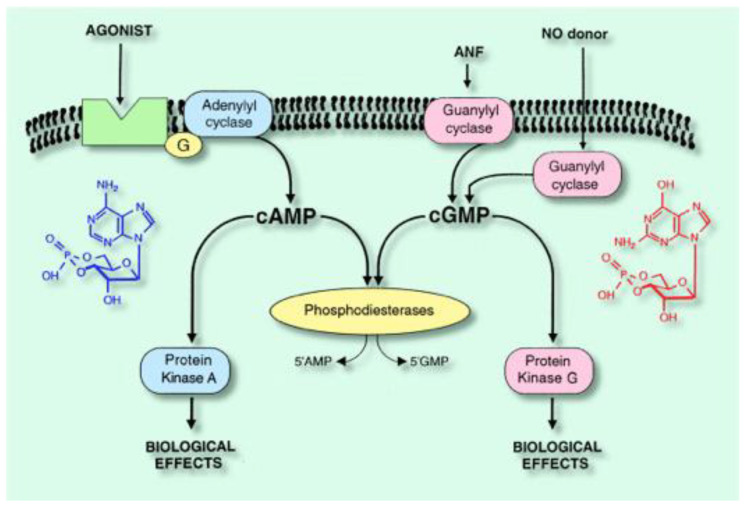
Intracellular signaling and cyclic nucleotide phosphodiesterases [2].

3′,5′- cyclic AMP (cAMP) plays a major role in the physiological control as well as in the pathophysiological control of tissue and cellular responses [1] (Figure 1) [2]. Its intracellular level depends:

- Firstly, on its synthesis by particulate adenylyl cyclases (pAC) governed by membrane receptors (for review: [3] or either by a soluble AC (sAC), [4];
- Secondly, its rapid degradation in 5′-AMP, by cyclic nucleotide phosphodiesterases (PDEs), allows the rapid restoration of the initial state of cellular cAMP level. Nevertheless, but interestingly, PDEs dysregulation plays a major role in many known, as well as unsolved, pathologies [5,6,7,8,9,10].

The PDEs represent a multigene superfamily, selectively hydrolyzing cAMP and /or cGMP (Figure 1). They are composed of 11 families representing more than 100 human variants. Among these, the biggest is the PDE4 family. It is constituted by four genes, giving rise to many proteins distributed differently cellularly and subcellularly, pointing out the diversity and the therapeutic potential of the PDE4 family [11]. 

### 1.1. The PDE4 Family

The PDE4 was first described as a PDE hydrolyzing selectively cAMP and thereafter was characterized by its specific sensitivity to the antidepressant compound rolipram (ZK 62711) and consequently named rolipram inhibited-cAMP-PDE (ROI-cAMP-PDE). Thus, differentiating it from the specific cAMP-PDE isolated from human platelets, named cGI-PDE, since this cAMP-PDE is inhibited by cGMP [12,13]. In another way, it was shown that rolipram is an antidepressant agent that binds with high affinity to the rat brain, opening the link between the high-affinity rolipram-binding site and the presence of PDE4 in the brain [14].

**Figure 2:**
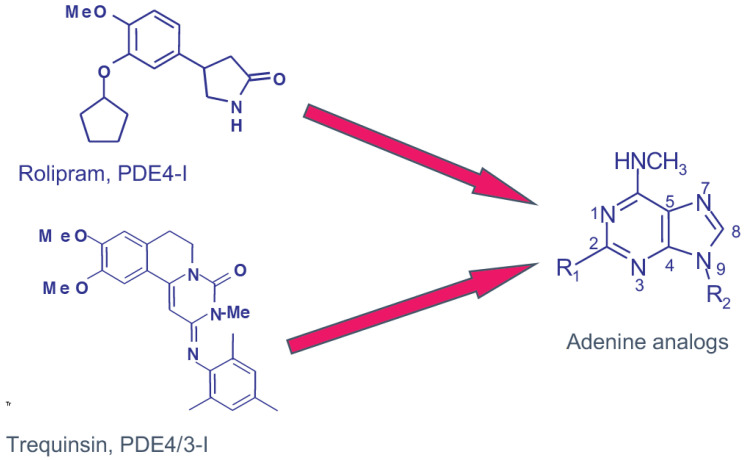
Design of adenine R_1_ and R_2_ as PDE4 inhibitors [15] based on rolipram and trequinsin structures identified as PDE4 inhibitors, with trequinsin inhibiting both PDE3 and PDE4 [13].

Consequently, together with Prof. C.G. Wermuth and Dr. J.J. Bourguignon, new PDE4 inhibitors [15] were conceived based on rolipram and trequinsin chemical structures opening a new field of research by designing R_1_ and R_2_ adenine nucleotides (Figure 2) [13,15]. 

Since PDE4 inhibitors act on inflammatory processes, mainly on oxygen-derived free radicals [16], and that moreover some failures (unsolved mortality) were obtained during the PDE3 inhibitor development, PDE4 research was preferentially developed. In another way, the cloning and expression of cDNA for a human low-K_m_, rolipram-sensitive cAMP phosphodiesterase was firstly reported in 1990 [17] as well as the structure of two rat genes coding for rolipram-sensitive cAMP-PDE [18]. Thus, pharmaceutical companies have conducted many studies to design and develop increasingly potent and specific PDE4 inhibitors (for review see: Houslay et al., 2005 [19]. Altogether, knowledge of the structures, properties, and functions of the PDE4 family has increased drastically. 

#### PDE4 Structure

**Figure 3:**
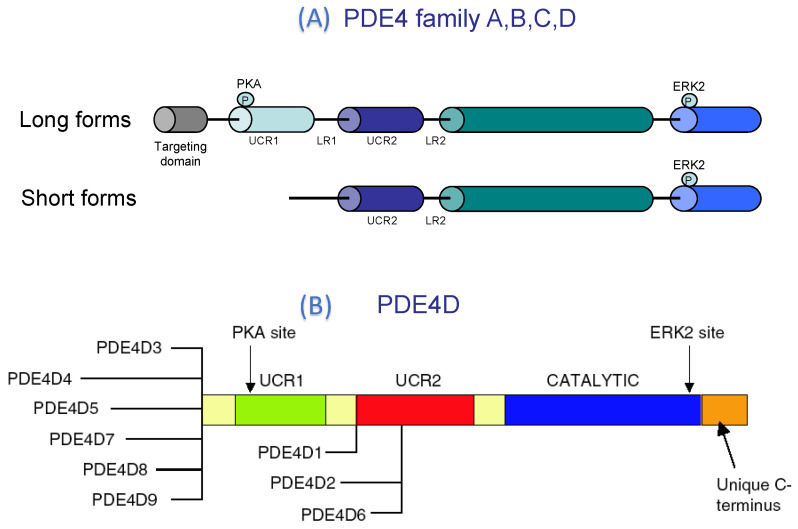
Structure of the PDE4 family. (**A**) Long forms of PDE4 contain both UCR1 and UCR2 and could be phosphorylated by PKA increasing PDE4 activity, whereas short forms lacking UCR1 could not be regulated by PKA and only exist as monomer [1]. (**B**) Diversity of PDE4D variants, Reprinted/adapted with permission from Ref. [24].

The PDE4 family, which exclusively hydrolyses the cAMP (K_m_ = 2–4 µM), is the most extensive family, coded by four genes, namely *PDE4A* to *PDE4D*. Each gene encodes multiple protein products generated by alternative splicing and/or by the use of multiple promoters (see Figure 3). More than 25 human isoforms of PDE4 are identified. The monomer of these proteins (from 50 to 125 kDa) is constituted by: (1°) a unique amino acid signature region called upstream conserved regions 1 and 2 (UCR1 and UCR2) which allows to classify PDE4 into long and short forms. The long PDE4 isozymes exhibit both UCR1 and UCR2, whereas short PDE4 isozymes lack UCR1 [20] (Figure 3). Since the UCR module provides dimerization, only long-form PDE4 splice variants containing UCR1 and UCR2 are dimerized [21]. UCR1 contains a PKA phosphorylation site that upon phosphorylation attenuates the ability of UCR1 to interact with UCR2 and thereby increases PDE4 activity; (2°) the catalytic domain, which constitutes the core of PDE, is a highly conserved region, with a 20–45% identity. It includes consensus metal-binding domains: two Zn^2+^-binding motifs (Hx3HxnE/D) and an Mg^2+^-binding motif, related to metal-ion phosphohydrolases [22], which are critical for the catalytic activity; (3°) the carboxyl-terminal end of the catalytic region contains an ERK-phosphorylation site. Its phosphorylation induces the activation of short forms of PDE4 (without UCR2) and inhibition of long forms of PDE4 [23] (Figure 3).

**Figure 4:**
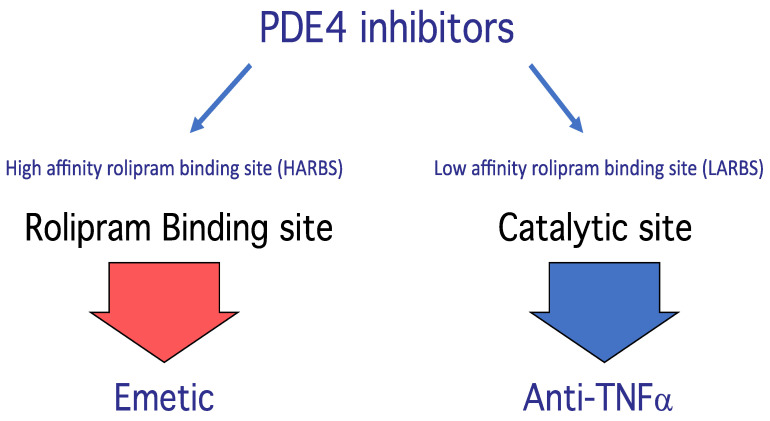
PDE4 inhibitors molecular mechanisms [24,25,26].

Rolipram, the prototype PDE4 inhibitor, binds to PDE4 at two sites, called the low-affinity rolipram binding site (LARBS) and the high-affinity binding site (HARBS) [12,24,25,26,27,28] (Figure 4). 

Interestingly, it has been shown that pregnancy induces a modulation of the PDE4-conformers ratio in human myometrium, designing LPDE4 as potential pharmacological targets for the design of new tocolytic treatments [29]. In contrast, HARBS mediates the antidepressant-like effects of PDE4 inhibitors [30]. Today, it seems that not enough people are interested in the biophysical concern of this two-site concept, but they merely suggest changes in spatial conformation inducing changes in cAMP hydrolytic activities.

## 2. PDE4 as Therapeutic Targets

Rolipram and Ro 20–1724 acting in the µmolar range were first identified as highly selective competitive inhibitors of PDE4 [1]. Subsequently, many specific PDE4 inhibitors acting in the nanomolar range were conceived as anti-inflammatory agents for asthma and COPD, as they reduced oxidative stress, TNF-α, and cytokine production. Roflumilast was the first PDE4 inhibitor developed on the market (called Daxas^®^) to treat COPD [31]. Lastly, apremilast (Otezla^®^), an oral PDE4 inhibitor, has received FDA approval to treat patients with active psoriatic arthritis and plaque psoriasis [32]. It is interesting to note that a different type of PDE4 inhibitor, classified as an allosteric inhibitor, has been developed opening up new perspectives. By closing UCR2 across one active site in the PDE4D dimer and by decreasing the catalytic activity of the second active site, it consequently reduces emetic potential [33]. Thus, the allosteric inhibitor of PDE4D compound BPN14770 is presently under development for enhancing memory. This compound acts newly on two different rolipram binding sites [34], re-opening questions about the concept of two affinity binding sites. 

Nowadays, there are different approaches under development targeting PDE4–protein interactions [35] allowing to locally and specifically alter PDE activity in the cell compartment of interest, named the signalosome, without acting on the whole cell or tissue. There may also be signalosome disruptors that affect interactions between PDEs and their regulatory partners [6,7]. In another way, the dissociation of the β1-adrenergic-receptor/PDE4 complex was reported as a novel mechanism of antagonist action [36]. Lastly, the identification of a PDE4-specific pocket design of selective inhibitors was reported that might improve the selectivity of PDE4 inhibitors [37]. 

Consequently, there are multiple pharmacological means to interact at the level of PDE4 changes inducing new specific and localized therapeutic approaches allowing less undesired effects. 

### 2.1. PDE4 in Cardiovascular Diseases

PDE4 participates in the control of cardiovascular function at the level of vessels and heart. 

#### 2.1.1. PDE4, and Vessel 

**Figure 5:**
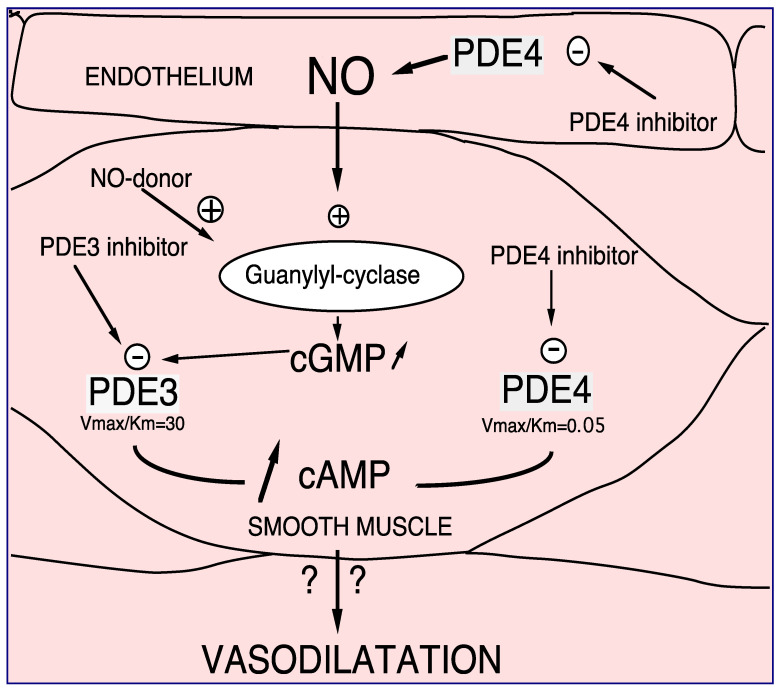
PDE3 and PDE4 cross-talk in vasodilation. NO or NO donor, by increasing soluble guanylyl cyclase, increases intracellular cGMP level. Therefore, this increase in cGMP induces preferentially PDE3 inhibition and consequently, an increase in cAMP in smooth muscle cells. In that way PDE4 might be able to hydrolyze cAMP in that way, PDE4 inhibitors inhibit PDE4 activity. In the presence of endothelial cells this effect is potentiated. Altogether, increases in cAMP altogether induces vasodilatation which might be potentiated in the presence of endothelium [38,39,40,41].

PDE4 has been characterized in human, rat, and bovine aortas [12]. It is clearly established that in vascular smooth muscle, PDE4 inhibitors control endothelium-dependent relaxation, opposite to PDE3 inhibitors or nitric oxide (NO) that per se relax the vessels [38,39,40]. This is related to the different kinetic properties of PDE3 and PDE4 (PDE3 K_m_ < PDE4 K_m,_ and PDE3 V_max/Km_ > PDE4 V_max/Km_) as illustrated in Figure 5. In the presence of endothelium, at low intracellular cAMP concentrations, firstly PDE3 hydrolyses cAMP; therefore, its specific inhibition, or the endothelial NO production, induces an increase in the cAMP level thus allowing PDE4 hydrolytic function. In that condition, the inhibition of PDE4 would be effective by increasing the cAMP level and would therefore relax the whole vessel. Altogether, at the level of vascular smooth muscle, we demonstrated the existence of cAMP/cGMP cross-talk mediated by PDE3 and PDE4 [40,41,42]. 

**Figure 6:**
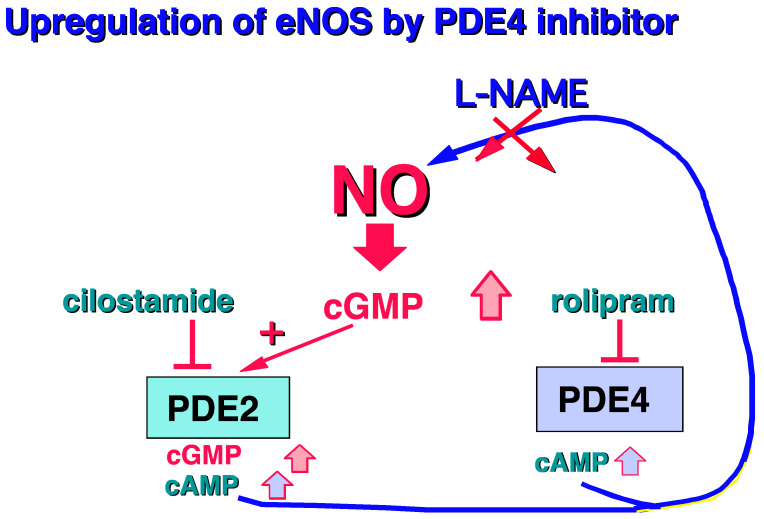
Increase in cGMP in human endothelial cells related to PDE4 inhibition [47].

Moreover, it should be noted that PDE4 is mainly present in human endothelial cells [43,44,45,46]. Surprisingly, in the presence of L-arginine, the PDE4 inhibitor, rolipram, is able to increase cGMP levels by upregulating the L-arginine/NO/cGMP pathway, revealing a cross-talk between PDE4 and cGMP regulation [47] (Figure 6).

In particular, PDE4 plays a role in the regulation of endothelial permeability [48]. Notably the PDE4D, by tethering the exchange protein activated by cAMP (EPAC)1 in a vascular endothelial cadherin-based signaling complex (VE-Cad), controls the cAMP-mediated vascular permeability [49]. It should be noted that heart failure in rat induces increases in the PDE4B protein and mRNA expressions in the aorta which could contribute to high blood pressure [50]. Lastly, it was shown that PDE4B, and not PDE4D, was upregulated in inflammatory cells from both experimental and human abdominal aortic aneurism, limiting the progressive increase in the aortic diameter without affecting the blood pressure. Rolipram has strongly mitigated the increase in vascular oxidative stress (superoxide anion) induced by angiotensin II [51]. Clinically, it is important to note that PDE4 inhibition reduces life-sustaining extracorporeal vascular permeability and improves microcirculation in an extracorporeal resuscitation rodent model [52].

#### 2.1.2. PDE4, and Heart

The cAMP in cardiac muscle is the second intracellular messenger mediating the positive inotrope effects of β-agonists [53]. Thus, PDE3 inhibitors, such as milrinone, SK, and F 94120, were described early in the early 1980s as “new cardiotonic drugs” [54,55]. Consequently, it was clearly established that the positive inotrope effect induced by PDE3 inhibitors was mediated by cAMP [56]. However, due to some mortality in chronic treatment related to tachyarrhythmia and tachycardia, milrinone prescription in humans is now only performed for a short period, beneficially inducing reduced inflammatory and apoptotic signaling [57]. At the same time, a rolipram-sensitive cAMP-PDE has been characterized in the heart. This was carried out by anion exchange chromatography performed on cardiac tissues from the rat heart ventricle [58], as well as from canine left ventricles [59] and from frog atrial fibers [60]. Furthermore, Komas et al., 1989 [55] isolated by chromatography the rolipram-inhibited cAMP-PDE (PDE4) from dog ventricular and sino-atrial node tissues, pointing out different inhibitory effects of specific inhibitors between the ventricle and sino-atrial node PDEs. Thus, the characterization of PDE4 in cardiac tissues raised the question of its implication in cardiac contraction. 

Studies performed on guinea pig cardiac ventricle showed that PDE4 might be implicated differently than PDE3 in the regulation of cardiac contraction, since in opposite to PDE3 inhibitor, PDE4 inhibitor did not per se increase cardiac inotropy, although PDE3 and PDE4 are both cytosolic and membrane bound [61,62]. However, interestingly, PDE4 inhibitors potentiate the effect of PDE3 inhibition, showing that in the presence of cyclic AMP-dependent positive inotropic agents, PDE4 inhibitors exert a positive inotropic effect which probably does not involve enhanced catecholamine release from sympathetic nerve endings [62]. This was confirmed more recently by Eschenhagen, 2013 [63] indicating that rolipram does not affect the positive inotropic effects from β1- or β2-adrenoceptor stimulation. This might also suggest that PDE4 acts in another cAMP compartment.

Thus, we firstly showed in canine and human, purified cardiac microsomal fractions, that PDE3 is associated with the microsomal membranes enriched in vesicles derived from T-tubule and junctional SR membranes; meanwhile, PDE4 is mostly associated with the enriched fraction of sarcolemma membranes, revealing different subcellular compartmentations for PDE3 and PDE4 [64]. To go further, we have canonically demonstrated that PDE4B and PDE4D are associated with the envelope of the nucleus isolated from human cardiac cells [65]. This has only been confirmed very recently by a pharmacological approach, demonstrating that PDE4 insulates a mAKAPβ-targeted PKA pool at the nuclear envelope [66]. In that way, Marco Conti and his colleagues showed that PDE4D isoforms are anchored by myomegalin colocalizing components of the cAMP-dependent pathway to the Golgi/centrosomal region of the cardiac cell [67]. Due to the different physiological and localized contributions of PDE4 in cardiac contraction, its physiopathological role in cardiac pathology could therefore be questioned. 

A study using PDE4D KO mice, clearly demonstrated that PDE4D deficiencies in the ryanodine receptor (RyR) complex promote heart failure and arrhythmias [68]. Furthermore, during cardiac hypertrophy in rats, there is a decrease in PDE4A, and PDE4B proteins and mRNA expressions, that accompany β-adrenergic desensitization, indicating some failures of PDE4s in cardiac pathologies [69]. However, it must be noticed that there is a five-fold higher amount of non-PDE4 activity in human hearts compared to rodents. As a result, only a restricted number of proteins could be phosphorylated in human cardiomyocytes, which would not affect overall cAMP signaling in the human heart [70]. 

PDE4D represents the main PDE4 isoform in the human heart and its expression is decreased in idiopathic dilated cardiomyopathy as well as in rats during early angiotensin II-induced cardiac hypertrophy, although total cAMP-PDE activity was increased [71]. In this way, PDE4D regulates the release of baseline sarcoplasmic reticulum Ca^2+^ and cardiac contractility, independently of L-type Ca^2+^ current, allowing for precise pharmacological control of cardiac contractility [72]. This could have a useful therapeutic implication for patients, since in failing human hearts, PDE4D co-immunoprecipitated with sarcoplasmic reticular (SR) calcium-ATPase type 2a but not with RyR2 [72]. Surprisingly, although PDE4D is part of a Ca_v_1.2 signaling complex, the inactivation of the pde4d gene had no consequence on basal or β-AR stimulated I_Ca_,_L_. In another way, studies performed in KO mice reveal that PDE4B plays also a key role during β-AR stimulation of cardiac function. As a result, PDE4B, by limiting the amount of Ca^2+^ entering the cell through the L-type Ca^2+^ channels, prevents Ca^2+^ overload and arrhythmias [73].

Altogether, decreases in PDE4D activity, which represents the main PDE4 isoform in human cardiac tissue, might favor idiopathic dilated cardiomyopathy and its deficiency in the RyR complex promotes heart failure and arrhythmias, whereas PDE4B prevents Ca^2+^ overload and arrhythmias. Interestingly, UCR1C is a new, long isoform PDE4 activator that alleviates cardiomyocyte hypertrophy [74]. Lastly, it was reported that decreases in PDE4 expression in the heart, related to fibroblast growth factor 23, increase the risk of cardiac arrhythmia [75].

### 2.2. PDE4 in Obesity

Obesity is considered the fifth highest cause of death worldwide by the World Health Organization and is associated with pathological conditions and diseases associated with obesity, including hyperlipidemia, heart diseases such as coronary artery disease (CAD) and myocardial infarction, stroke, type 2 diabetes (T2D), hypertension, cancers, low-grade and chronic inflammation, fatty liver disease, osteoarthritis, respiratory problems, and neurodegenerative diseases [76]. 

Obesity is defined by the body mass index (BMI). It is calculated as weight in kilograms divided by height in meters squared, rounded to one decimal place. Obesity in adults (>20 years old) was defined as a BMI greater than or equal to 30. The prevalence of obesity was 39.8% and affected about 93.3 million US adults in 2015~2016 [77]. 

Therefore, it was pointed out that caloric restriction might extend lifespan and that the metabolic effect of resveratrol, a mimetic of caloric restriction, might be mediated by inhibiting cAMP phosphodiesterases [78]. In that way, alterations in PDE activities were firstly reported in omental and subcutaneous adipose tissues in human obesity, i.e., in omental and subcutaneous adipose tissues, it was a significant negative correlation between PDE4 and BMI [79].

Thus, the involvement of PDE4 in obesity has been demonstrated since, under submaximal β-adrenoceptor stimulation of brown adipocytes, the PDE4 inhibitor alone may increase lipolysis [80]. Therefore, it was reported that the PDE4 inhibitor, roflumilast, might be superior to metformin in weight loss in obese women with polycystic ovarian fibrosis [81]. In this way, PDE4B-KO mice had reduced adiposity and adipose-induced inflammation induced by a high-fat diet, highlighting the involvement of PDE4B in adiposity [82].

A study of the Saudi population found that PDE4D polymorphism (rs295978) was not associated with a risk of obesity and did not affect the blood lipid profile [83]. Nevertheless, the allosteric PDE4D inhibitor, compound D159687, induces weight loss and fat mass loss in older mice without changing lean mass and physical and cognitive function [84]. Thus, we proposed that PDEs and in particular PDE4 participate in the metabolic syndrome by regulating the AMP-activated protein kinase (AMPK) [85]. In this way, it is noteworthy that the effect of roflumilast depended on the activation of the metabolic regulator AMPKα. Consequently, suppressed adipogenesis and promoted lipolysis in cell culture and mouse models suggest the therapeutic potential of roflumilast in obesity-related diseases [86].

Altogether, PDE4 regulation seems to be implicated in obesity at the level of PDE4B and PDE4D, and an allosteric inhibitor of PDE4D would be effective in the loss of fat mass without changing cognitive function.

### 2.3. PDE4 in Diabetes

The global prevalence of diabetes among 20- to 79-year-olds is estimated to be about 8.8% in 2015, or 415 million, and approximately 10.4% in 2040, or 642 million [87]. Diabetes is related to alterations in glucose homeostasis governed by insulin. The failure to maintain glucose homeostasis underlies both type 1 diabetes (T1D) and type 2 diabetes (T2D). T1D is an autoimmune disease that originates when β-cells that produce insulin are destroyed. T2D is characterized by insulin resistance and the progressive loss of β-cell function. Although these two forms of diabetes are fundamentally very different, β-cell failure and death play a key role in the pathogenesis of both diseases, leading to hyperglycemia resulting from a reduced capacity to produce insulin [88]. The diagnosis of diabetes and its severity may be determined by measuring blood glycated proteins such as hemoglobin A1c (HbA1c) and glycated albumin (GA) [89]. In the 2000s, it was proposed that inhibiting PDE4 during diabetes may be beneficial against hyperglycemia, oxidative stress, and the production of TNF-α and NFκB [90]. In this way, it has previously been reported that insulin-secreting cells and Langerhans islets contain PDE4 [91]. PDE4C is the main PDE4 subtype expressed at the mRNA level in isolated rat islets. Interestingly, silencing the PDE4C as well as the specific PDE4 inhibitors, roflumilast and compound L-826,141, significantly increases glucose-dependent insulin secretion [92]. PDE4 seemed active only after stimulation with glucose, suggesting some interaction between glucose and PDE4 [93]. Following this, a study in mice showed that roflumilast improves glucose tolerance and insulin sensitivity [93].

Recently, TAK-648, a PDE4 inhibitor, with demonstrated preclinical anti-diabetic properties, has been developed for human T2D (dose prediction using HbA1c results from a db/db mouse study), opening the way for new therapeutic approaches in diabetes [94]. Interestingly, a recent review on PDE4 inhibitors in diabetic nephropathy highlights this project [95] as well as a review on PDE4 inhibitors in prediabetic patients [96].

### 2.4. PDE4 and Ulcerative Colitis and Crohn’Disease

Crohn’s disease is a chronic inflammatory condition of the gastrointestinal tract. It can affect any part of the gastrointestinal (GI) tract, but ulcerative colitis affects only the colon. Additionally, while Crohn’s disease can affect all layers of the bowel wall, ulcerative colitis (UC) only affects the lining of the colon. Both have been together classified as inflammatory bowel diseases (IBD). In the United States, it is currently estimated that about 1.5 million people suffer from IBD, causing considerable suffering, with a prevalence of Crohn’s disease (CD) of 201 per 100,000 population, UC is being equally prevalent [97]. 

Because Crohn’s disease is mainly a chronic inflammation, the inflammatory cytokine productions in intestinal biopsies have been studied in relation to the pathological grade. This study originally shows that TNF-α and IL-1β are significantly increased in endoscopic biopsies, as well as for TNF-α, IL-1β, and IL-6 in the individual culture supernatant of intestinal biopsies, opening a new way of investigation [98]. Thus, the effect of pentoxifylline was investigated on intestinal inflammation in IBD, showing that pentoxifylline downregulates in vitro TNF-α and IL-1β production by PBMCs and by intestinal organ cultures from patients with Crohn’s disease and ulcerative colitis [99]. Because pentoxifylline, acting on TNF-α and IL-1β, is a non-selective PDE inhibitor [100], PDE activities were investigated in the human normal mucosa and inflamed mucosa of patients with Crohn’s disease. These studies revealed an increase in the % of PDE4 activity (from 42% to 72.5 %, up by *p* < 0.05%) suggesting that the specific PDE4 inhibitor may be effective in Crohn’s disease [101]. In that way, the use of OPC-6535 (tetomilast) was suggested for the treatment of a variety of oxidative inflammatory intestinal disorders with an abnormal mucosal barrier such as inflammatory bowel disease [102]. As a result, tetomilast was developed by OTSUKA (phase III) to investigate the treatment of UC [103]. Furthermore, it has been clearly reported that the PDE4 inhibitor, rolipram, prevents and reduces experimental colitis in mice and suppresses TNF-α levels in colonic tissues [104]. Interestingly, rolipram is shown to be superior to methylprednisolone in preventing late collagen deposition [105].

Overall, today, no PDE4 inhibitor has been developed for IBDs, although it appears to be more effective than methylprednisolone [105]. This may be possible because of limitations related to side effects of PDE4-Is, such as nausea and vomiting, see Ref. [9]. Today, with the new designs of PDE4-I, this should be re-investigated. In this way, it has recently been suggested that inhibition of PDE4 (notably by apremilast) may be a new therapeutic avenue for IBD [106]. Recently, a clinical study on the use of PDE4-I in psoriatic arthritis and IBD suggested that the use of apremilast should be studied merely in enteropathic spondylarthritis [107]. However, very few studies have focused on characterizing the altered PDE4 subtype in IBD. To go further, it would be of interest to develop either the selective PDE4 inhibitor acting specifically on the PDE4 subtype implicated in the disease or to investigate a new kind of PDE4-I such as allosteric PDE4-I [6,7]. A better prognosis of patients with Crohn’s disease has been recently proposed that combines endoscopic and radiological healing, which would favor the development of PDE4-Is [108].

### 2.5. PDE4 and Osteoporosis

Osteoporosis is a common disease characterized by reduced bone mineral density (BMD) and increased risk of fragility fractures, which is usually caused by osteoclasts, whereas osteopenia corresponds to a lower bone density than normal and represents the stage before osteoporosis. By 1970, no medications against osteoporosis were being investigated. At the time, fractures were not even recognized as an illness but were considered part of normal aging [109]. Nonetheless, osteoporosis is a public health issue worldwide, affecting over 200 million people. An estimated 30% to 50% of postmenopausal women have this disease. The prevalence (June 2009 to June 2010) of osteoporosis and osteopenia in healthy active workers is 18% [110]. However, it must be pointed out that osteoporosis is an age-associated disease. In the Eastern Mediterranean region, the overall pooled prevalence of osteoporosis was 24.4%, pointing out the necessity of investigating therapeutic approaches [111]. 

In that way, a study showed that denbufylline, an inhibitor of PDE4 [112,113], was shown to increase the number of mineralized nodules and decrease the number of osteoclast-like cells, suggesting that it should be a therapeutic drug for bone loss [114]. Therefore, this study was reinforced by studying the effect of a new PDE4 inhibitor, XT-44, on mineralized nodule formation, as well as in vivo in ovariectomized female Wistar rats. XT-44 stimulated the formation of mineralized nodules, while it inhibited the formation of osteoclast-like cells in mouse bone marrow culture. Interestingly, in ovariectomized female rats, 1 mg/kg per os (every 2 days for 8 weeks) increased bone mineral density, indicating that XT-44 may be effective in osteoporosis treatment [115]. 

In addition, pentoxifylline and rolipram have been shown to increase bone mass in normal mice mainly primarily by accelerating bone formation [116,117]. This was confirmed in adult ovariectomized rats in which rolipram at 0.1–0.6 mg/kg dose levels prevented, while at 1 mg/kg restored ovariectomy-induced cancellous and cortical bone loss [118]. Thus, studies performed on human culture cells showed by chromatographic separation that SaOS-2 human osteosarcoma cells contain PDE4 activity, as well as PDE1 and PDE7 activities, PDE4 activity being undetectable in cultured normal human osteoblasts. Nevertheless, transcripts of PDE4A and PDE4B were characterized in both kinds of human cells. Furthermore, only in sarcoma cells does rolipram raise the cAMP level according to the dose. 

This human cell study, combined with previous studies [104,105,106,107], suggests that PDE4 could be a new target in the treatment of osteoporosis [119]. Therefore, since dexamethasone glucocorticoid is known to enable the development of osteoporosis, its effect was investigated on the expression of PDEs in SaOS-2 cultured sarcoma cells. Thus, in agreement with a decrease in PDE4 activity, and rolipram sensitivity, a 50–70% decrease in the mRNA of PDE4A and PDE4B subtypes, associated with a reduction in PDE4A4 and PDE4B1 isoforms were reported following dexamethasone treatment.

In total, these data point to the participation of PDE4 in osteoporosis [120]. Furthermore, it was shown that PDE4 inhibitor enhances PGE2-induced growth arrest of osteoclast formation. It has been found that the PDE4 inhibitor in one way promotes the formation of osteoblasts, and in another way, inhibits the formation of osteoclasts, both of which might may increase bone formation [121].

Although it was shown that PDE4-Is are effective in decreasing osteoporosis, this potential therapeutic approach always seems to not be recognized for osteoporosis [122]. Hopefully, very recently, Porwal et al. 2021 suggested repurposing the potential of PDE inhibitors in osteoporosis [123].

### 2.6. PDE4 and Malignancies 

According to MedlinePlus Medical Encyclopedia, “the malignancy” refers to the presence of cancerous cells that have the ability to spread to other sites in the body (https://medlineplus.gov/enc/article/002253.htm, accessed on 13 November 2020). The global cancer incidence and mortality in 2020 were estimated at 19.3 million, almost a million new cases of cancer and 10 million deaths from cancer. The global cancer burden is expected to be 28.4 million cases in 2040 [124].

The use of PDE4-Is in various developing cancers was first investigated in various cancer cell lines as well as on cancer tissues [125,126,127]. Therefore, it was shown that PDE4 protein and mRNA up-regulations are associated in vivo with human endothelial cell proliferation and angiogenesis [45,128] as well as in vivo cancer development in mice [129]. Similarly, there has been reported a phosphodiesterase 4B-dependent interplay between tumor cells and the microenvironment regulating angiogenesis in B-cell lymphoma [130]. Since the PDE4-I, piclamilast, was able to potentiate the cyto-differentiating action of retinoids in myeloid leukemia cells [131], we investigated PDE activity and expression in retinoic acid-resistant cell lines in acute promyelocytic leukemia and showed an increase in PDE4 activity, accompanied by an increase in PDE4D expression [132]. In another area, studies performed in mice, rats, and human tissue revealed that PDE4B protects colon adenomas and is inactivated by epigenetic silencing in colon cancer [133]. 

Interestingly, the focal cAMP reduction achieved by forced expression of PDE4A1 in the cortex of neurofibromatosis-1 (NF1) genetically engineered mouse (GEM) model is sufficient to induce gliomagenesis in a mouse model of NF1, and conversely, rolipram dramatically inhibits in vivo optic glioma growth and tumor size in the model [134]. In ovarian cancer development, roflumilast mediates in vivo tumor inhibition by up-regulating mitochondrial ferritin in nude mice [135]. In another area, PDE4D7 was found to be a marker for the potential diagnosis of prostate cancer [136]. Thus, PDE4 inhibition decreases the malignant properties of DLD-1 colorectal cancerous cells by repressing the AKT/mTOR/Myc cancerous signaling pathway [137]. Conversely, PDE4 and EPAC1 synergistically promote rectal carcinoma via the cAMP pathway in human tissues, highlighting the PDE4 implication in colorectal cancer [138]. It must be pointed out that PDE4 inhibition by rolipram induced analgesia in bone cancer pain by inhibiting the activation of the spinal astrocytes and is associated with the downregulation of spinal IL-1β, IL-6, and TNF-α expressions [139]. 

Some PDE4-Is were recently considered cancer therapeutics, including apremilast [140]. In this area, it should be noted that patients with psoriasis may present a high risk of melanoma and hematological cancers, regardless of their psoriasis treatment [141]. Lastly, the participation of PDE4 subtypes in various cancers has been reviewed by Hsien Lai et al., 2020 [142].

### 2.7. PDE4 and Fatty Liver Disease

Fatty liver disease, currently named nonalcoholic fatty liver disease (NAFLD) according to the practice guidance commissioned by the American Association for the Study of Liver Diseases (AASLD), is defined by: (1) evidence of hepatic steatosis (HS), either by imaging or histology, and (2) lack of secondary causes of hepatic fat accumulation such as significant alcohol consumption, long-term use of a steatogenic medication, or monogenic hereditary disorders [143]. The overall global prevalence of NAFLD diagnosed by imaging is about 25.24% (95% CI, 22.10–28.65) [144]. NAFLD and alcoholic liver disease (ALD) are the leading causes of liver-related morbidity and mortality and are important causes of liver transplantation [145]. Using bile-duct ligation, as a cholestatic liver injury model, Gobejishvili and colleagues 2013 [146] demonstrated that induction of hepatic PDE4A, B, and D plays a causal role in the development of liver injury and fibrosis. In addition, roflumilast PDE4-I improved glucose tolerance, reduced insulin resistance, and decreased steatohepatitis in mice, increasing the cellular respiratory capacity of hepatocytes [93]. Interestingly, Vonghia et al., 2019 [145] mentioned that the compound tipelukast, also known as MN-001, designed as a leukotriene receptor antagonist, orally bioavailable, being anti-fibrotic and anti-inflammatory in pre-clinical models, interestingly, inhibits PDE3 and PDE4. However, a lack of efficacy of a PDE4-I in phase 1 and 2 trials of patients with non-alcoholic steatohepatitis has already been shown [147]. 

Overall, these data suggest that today PDE4-Is have failed in the treatment of NAFLD but have induced interesting positive data regarding ALD treatment. Thus, it has been shown that altered PDE4B plays a critical role in alcohol-induced steatosis [148]. A recent review designs PDE4 inhibition as a therapeutic target for ALD as much as, expression of the PDE4 subfamilies is significantly up-regulated in conjunction with markedly decreased cAMP levels in hepatic tissues of patients with severe ALD [149]. It should be noted that inhibition of PDE4 decreases ethanol intake in mice, extending the contribution of PDE4 to liver diseases [150]. Thus, contrary to ALD, one could speculate that liver PDE4 is not sufficiently induced in NAFLD to demonstrate changes in PDE4 expression and efficiency of PDE4-Is. Nonetheless, overexpression of PDE4 in the mouse liver is sufficient to trigger NAFLD and hypertension and can be avoided and even reversed by roflumilast PDE4-I [151]. A very recent study has supported the clinical use of a novel liposomal rolipram formulation to reduce emesis [152].

### 2.8. PDE4 and Depression

Major depression is a common illness that severely limits psychosocial functioning and diminishes quality of life. In 2008, the WHO ranked major depression as the third cause of burden of disease worldwide and projected that the disease will rank first by 2030, the 12-month prevalence of major depressive disorder (MDD) being approximately 6.6%, and the lifetime risk being 15–18% [153]. 

The potential antidepressant activity of rolipram, as a cAMP phosphodiesterase inhibitor, was canonically demonstrated in mice by Wachtel H, 1983 [154]. In that way, rolipram was first reported as a specific PDE4-I [12]. Rolipram was shown to have antidepressant-like effects on behavior maintained by differential reinforcement of low rat response [155] and to facilitate the establishment of long-lasting long-term potentiation and improve memory [156]. In PDE4D knockout (PDE4D^−/−^) mice, the loss of PDE4D in the cerebral cortex and hippocampus, interestingly, induces an antidepressant-like effect on behavior and reduces sensitivity to rolipram. Furthermore, rolipram potentiated isoproterenol-induced cyclic AMP formation only in the PDE4D^+/+^ mice. Interestingly, the PDE4D-regulated cyclic AMP signaling may play a role in the pathophysiology and pharmacotherapy of depression [157].

According to a review on CNS and PDEs, it is pointed out that PDE4A, 4B and 4D may participate in depression [158]. Mice deficient in the PDE4B gene (PDE4B^−/−^) displayed long-term depression as compared to wild-type littermates [159]. In this case, the use of novel allosteric small-molecule activators of the long-form PDE4 would be helpful [160]. However, the use of a new isoform selective technological approach, reducing dramatically the catalytic enzyme activity of PDE4B1, (mainly present in hippocampal CA2 and CA3 regions) being more specific than the PDE4B gene knockouts, allows seeing increased activity in these mice without major changes in associative learning or other behavior. These data might contribute to the development of PDE4-Is [161]. The development of new technologies has allowed demonstrating the implication of PDE4 in stress-induced depressive-like behaviors, since the knockdown of long forms of PDE4D4 and PDE4D5 in the mouse prefrontal cortex alleviates stress-induced depression and chronic unpredictable stress-induced depressive-like behaviors in mice [162]. In that way, roflumilast, a more potent and selective PDE4 inhibitor than rolipram, improves memory in rodents at non-emetic doses, pointing out PDE4 as a possible therapeutic target in cognition, demonstrating the therapeutic potential of PDE4 inhibitors in the brain [163].

Therefore, strategies to develop antidepressant PDE4 inhibitors is to develop a new generation of PDE4 inhibitors which act specifically on long PDE4 isoforms. They might also be capable of targeting the interactions with their signaling “partner” proteins, such as RACK1, β-arrestin, and DISC1 in order to affect cAMP levels in specific cellular compartments [164]. In addition, the understood mechanism of PDE4D involvement in Alzheimer’s disease opens up new therapeutic approaches that may focus on the activation status of PDE4 [165]. Interestingly, the identification of diagnostic markers MDD (761 differentially expressed genes closely related to MDD) has recently been validated in total blood samples. The gene set enrichment analysis suggested that the tumor necrosis factor signaling pathway, toll-like receptor signaling pathway, apoptosis pathway, and NFkB signaling pathway are all crucial in the development of MDD [166] in accordance with known PDE4-dependent signaling. Recently, these approaches were conducted in a pre-clinical study using roflumilast in transgenic APP/PS1mice [167]. Since all these pathways concern PDE4 participation, it would be very interesting to include PDE4 as a diagnostic marker in MDD. Furthermore, these diagnostic markers will help in the development of specific PDE4 inhibitors targeting MDD with lower adverse effects. In that way, a recent review on PDE4 inhibition in the CNS, suggests new approaches focused on targeting PDE4 conformational states and on influencing the binding affinity of PDE4 subtype inhibitors [168]. 

### 2.9. PDE4, and COVID-19

As previously [169] stated, during the 1990s, PDE4 might participate in viral infections, since the PDE4 inhibitor, rolipram, was canonically shown to inhibit human immunodeficiency virus-1 (HIV-1) replication, and to decrease HIV-1 p24 antigen production in acutely HIV-1 infected PBMCs [170]. This rolipram inhibitory effect on HIV-1 replication in Jurkat and primary T-cells induced by T-cell activation has been confirmed and extended to the production of TNF-α, NFκB, and NFAT activation [171]. Beavo and colleagues showed that infection of CD4+ memory T-cells by HIV-1 requires the expression of PDE4, and that rolipram abolishes HIV-1 DNA nuclear import in memory T cells, pointing out the important contribution of PDE4 in viral infection [172]. Roflumilast inhibits respiratory syncytial virus infection in differentiated human bronchial epithelial cells [173]. However, in the human airway smooth muscle infected by rhinovirus, a non-enveloped RNA virus, surprisingly, inhibition of PDE4 did not overcome cytokine induction [174], opening the hypothesis that the viral envelope is a requisite for cytokine induction and PDE4-I effectiveness. Interestingly, it has been reported that cAMP produced in Tregs is implicated in suppressing the activation and expression of the HIV-1 gene in vivo in humanized mice [175]. Since a relationship between HIV-1 and PDE4 was clearly established, one could wonder whether COVID-19 might modify PDE4 regulation. 

Interestingly, a paper published in Nature Medicine (2005), demonstrated that angiotensin-converting enzyme 2 (ACE2) plays a strategic role in SARS-CoV-2-induced lung injury. As expected, the injection of SARS-CoV-2 spikes into mice worsens acute lung failure in vivo which can be alleviated by blocking the renin–angiotensin pathway governed by ACE2 [176]. Furthermore, it has been reported that Ang-II, an AT_1_R agonist, induces PDE4 up-regulation, with a 44% increase in the PDE4A protein, mediating cascade inflammation and oxidative stress. Altogether, this opens a new way of PDE interaction with the renin–angiotensin system [71]. More recently, relationships between PDE4, and cytokine storm were discussed for COVID-19 [177,178,179,180]. In this way, a recent in vitro study (phase III) performed with the inhaled PDE4 inhibitor, tanamilast (CHF6001), showed that it blunts proinflammatory dendritic cell activation by SARS-CoV-2 ssRNAs [181].

Altogether, these data reported for COVID-19 suggest the hypothesis that a PDE4 inhibitor might alleviate both viral infection and tissue inflammation induced by SARS-CoV-2 [169]. 

## 3. Conclusions and Perspectives

**Figure 7:**
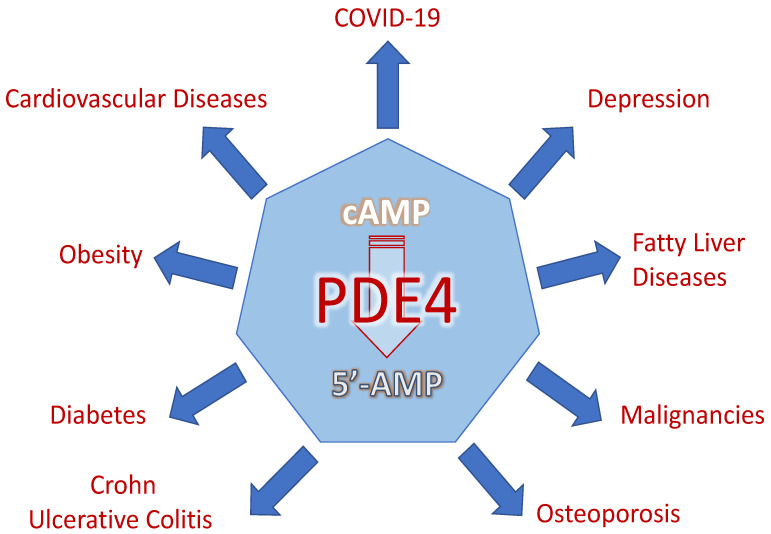
PDE4, a therapeutic target in various human diseases.

Altogether, PDE4s are implicated in various human pathologies, such as cardiovascular diseases, obesity, diabetes, ulcerative colitis and Crohn’s, osteoporosis, malignancies, fatty liver disease, and depression, as well as COVID-19 (Figure 7). Given that PDE4 is an intracellular target, it is possible that at the same time PDE4 may participate differently in two or more diseases. Therefore, there has recently been a reported risk of malignancy, and recurrence associated with psoriasis and its treatments. This was pointed out for PDE4-Is in patients with a history of cancer, suggesting the necessity of long-term studies focused on safety surveillance [182]. In another way, the neuroprotective effect of a PDE4 inhibitor (FCPR03) against oxygen-glucose deprivation has been recently related to the activation of nuclear factor erythroid 2-related factor 2 (Nrf-2)/heme oxygenase-1(HO-1) attenuating ROS production and consequently endoplasmic reticulum stress and apoptosis in neurons, allowing to decipher its molecular mechanism [183].

To further explore the therapeutic potential of PDE4-Is, different strategic approaches could be used to reduce adverse effects. Since PDE4 could act in interactome signaling, it should be possible to act on the protein–protein interactions governing PDE activity in various cell compartments [184,185]. This was illustrated for a multifunctional docking site on the catalytic unit of PDE4 which is used by several interaction partners [186]. Of interest, a recent review analyzed in detail the advances in knowledge and in patent developments. It originally reports the diverse ways to interact with PDE signals directly or not, in order to overcome PDE dysregulations associated with various dysfunctions [187]. Nevertheless, it should be pointed out that other PDE families present in the vicinity of PDE4 might interfere with PDE4 responses such as PDE3 [39,41], PDE2 [45,188], as well as PDE1 and PDE5 [71].

The present paper reports the implication of dysregulation of PDE4 activity in various pathologic fields pointing out the effectiveness of PDE4 inhibitors as well as the increases in the knowledge of PDE4 functions related to their multiplicity at cellular, subcellular, and molecular levels. This introduces new approaches to powerful, selective, and effective treatment, with lesser adverse effects, opening up a new generation of more suitable PDE4 inhibitors. This was just newly discussed and reviewed for clinical implications of PDE4-Is [189].

## Data Availability

Not applicable.
